# Cytoplasmic Electric Fields and Electroosmosis: Possible Solution for the Paradoxes of the Intracellular Transport of Biomolecules

**DOI:** 10.1371/journal.pone.0061884

**Published:** 2013-04-16

**Authors:** Victor P. Andreev

**Affiliations:** 1 Department of Psychiatry and Behavioral Sciences, University of Miami Miller School of Medicine, Miami, Florida, United States of America; 2 Department of Biochemistry and Molecular Biology, University of Miami Miller School of Medicine, Miami, Florida, United States of America; 3 Center for Computational Sciences, University of Miami, Miami, Florida, United States of America; SUNY Downstate MC, United States of America

## Abstract

The objective of the paper is to show that electroosmotic flow might play an important role in the intracellular transport of biomolecules. The paper presents two mathematical models describing the role of electroosmosis in the transport of the negatively charged messenger proteins to the negatively charged nucleus and in the recovery of the fluorescence after photobleaching. The parameters of the models were derived from the extensive review of the literature data. Computer simulations were performed within the COMSOL 4.2a software environment. The first model demonstrated that the presence of electroosmosis might intensify the flux of messenger proteins to the nucleus and allow the efficient transport of the negatively charged phosphorylated messenger proteins against the electrostatic repulsion of the negatively charged nucleus. The second model revealed that the presence of the electroosmotic flow made the time of fluorescence recovery dependent on the position of the bleaching spot relative to cellular membrane. The magnitude of the electroosmotic flow effect was shown to be quite substantial, i.e. increasing the flux of the messengers onto the nucleus up to 4-fold relative to pure diffusion and resulting in the up to 3-fold change in the values of fluorescence recovery time, and therefore the apparent diffusion coefficient determined from the fluorescence recovery after photobleaching experiments. Based on the results of the modeling and on the universal nature of the electroosmotic flow, the potential wider implications of electroosmotic flow in the intracellular and extracellular biological processes are discussed. Both models are available for download at ModelDB.

## Introduction

The majority of the studies of the intracellular electric fields discuss the role of membrane potential and electric fields across cellular and nuclear membranes as reviewed in [1]–[2]. The electric field in the cytoplasm is usually ignored based on the reasoning that “high ionic strength and electrical conductibility of physiological media do not allow a significant electric field to be sustained at distances greater than 1 nm (the Debye length) from the originating charge distribution” [1]. This reasoning, however, ignores the fact that electric field and electric current do exist in a conductor connected to electric source, e.g. battery or a generator. Similarly, the biological cell is an active device which generates ion gradients with the help of ion pumps (carrier protein coupled to a source of metabolic energy such as ATP hydrolysis). These ion gradients allow the passive transport of ions through the ion channels of the cellular membrane [3]. Importantly, “when ion pump and/or ion channel activity is asymmetrically distributed over the plasma membrane, the cell may be able to drive ionic fluxes through itself” and “behaves as a miniature electrophoretic chamber” [4]. This self electrophoresis principle was developed as early as 1966 [5], visualized in the meroistic ovary of an insect [6]–[8], further investigated and reviewed in [8], [4]. The plethora of possible cytoplasmic electric field and electric current configurations were discussed, depending on the distribution of ion pumps and ion channels, position of nucleus (in or out of main transcytoplasmic flux) and type of nuclear envelope [4].

The recent development of nanoparticles filled with the voltage-sensitive fluorescent dye, allowed the direct measurement of the intracellular electric fields in the cytosolic and membrane regions of living cells (astrocytes) [9]. Importantly, strong electric fields (5·10^5^–3·10^6^ V/m) were observed not only in the membrane and organelle regions but in the cytoplasm. The strongest electric fields were observed in the vicinity of mitochondria (inner mitochondrial membrane is known to have high electric potential of −150 mV [10]–[11]), however it was still quite strong (5·10^5^ V/m) at the distance of several micrometers away from mitochondria [9].

The role of the cytoplasmic electric fields in the intracellular transport of proteins was investigated in two recent papers [12]–[13]. These papers argue that the transport of the messenger proteins from the cellular membrane to the nucleus cannot be due to diffusion alone since it is slow and “would result in broad dispersion of information in cytoplasm”. Based on the observations of [14]–[15] it was assumed that the movement of the messenger proteins was not “facilitated by interactions with microtubules and microfilaments” but was due to the free movement in the cytosol. As a mechanism of directed motion of phosphorylated messenger proteins, the authors of [12]–[13] suggested the coulomb interactions of the negatively charged messenger proteins and intra-cytoplasmic electric field generated by the positively charged “outer rim of the nuclear membrane” [12]. The experimentally observed distributions of the fluorescently labeled RAF, MEK, and ERK proteins in the culture of human mammary epithelial cell were in better agreement with the theoretically predicted protein distributions with the above electrostatic forced transport than with the distributions predicted for the case of pure diffusion [13].

The simulations of [12]–[13] were based on the assumption of the positively charged nucleus membrane of the cell (see Table 1 and eq. 4 of [12]). However, the validity of this assumption is far from obvious. The comprehensive review [2] of the electric properties of nuclear envelope (NE) presents the data from thirteen papers on the microelectrode measurements obtained in intact cell nuclei of various species. In ten of the above 13 papers, the NE potential was reported negative, starting at relatively low potential of −0.3 mV in salivary gland cells [16] to substantial negative potential of −33 mV in HeLa cells [17]. Only one of the studies [18] reported zero NE potential in oocytes, while two of the reviewed studies presented data on the nuclear envelope resistance and no data on the NE potential [19]–[20]. None of these 13 studies reported positive potential of the nucleus. Based on these observations, and observations of [21] on the isolated nuclei, the authors of [2] concluded that “cell nucleus could be considered as a “negatively charged sink” that is in continuous interaction with the positive charges originating from either macromolecules imported into the cell nucleus through nuclear pore complexes or from inorganic cations (such as Ca^2+^ and K^+^) that could diffuse across NE via ion channels”. The assumption of the positively charged nucleus membrane used in [12]–[13] is in contradiction with this conclusion.

**Table 1:** Parameters used in the simulations

Parameter	Value	Unit	Description	References
a, h	10^−5^, 10^−5^	m	Cell radius and height	[12]–[13]
c	3•10^−6^	m	Nucleus radius	[12]–[13]
d, e	0, 5 •10^−6^	m	Nucleus location (r; z)	[12]–[13]
ε_1_, ε_2_	60, 120	Dimensionless	Relative dielectric permittivity of the cytoplasm and nucleoplasm	[37]
σ_1_, σ_2_	0.25, 0.5	S/m	Conductivity of the cytoplasm and nucleoplasm	[37]
*V_0_*	−0.033	V	Electric potential of the nucleus	[2], [17]
*J_n_*	1500	A/m^2^	Current densities, top and bottom of the cylinder (cell model)	[6], [s5–s9]*
*η*	0.002	Pa•s	Viscosity of cytoplasm	[39], [s24,s28,s32]
*ζ*	−0.05	V	Zeta-potential of cellular membrane	[s15,s16]
*D_C_*, *D_N_*	14•10^−12^,0.04•10^−12^	m^2^/s	Diffusion coefficients of the messenger protein in the cytoplasm and nucleoplasm	[s46,s47], [s48]
*w_L_*, *z_L_*	2•10^−6^, 10^−5^	M	FRAP laser beam radii in radial and axial directions	[44]
*K_L_*	6	Dimensionless	Bleach efficiency	[44]

* [s5], [s9], etc–means reference from the (“Methods S1. Parameters Justification” file).

Following [2], we assume that cell nucleus is negatively charged at least in some cells. Therefore, it is important to understand how the negatively charged messenger proteins can travel to the negatively charged nucleus against the electrostatic repulsion. In this paper, we suggest electroosmotic flow as a solution of this apparent paradox.

Electroosmotic flow is a well known effect, described by Smoluchowski as early as 1914 [22] and caused by the presence of the electrical double layer, formed at the fluid/solid or fluid/membrane interface, and by the action of the electric field parallel to this interface. The flow of the fluid results in the movement of the particles immersed in the fluid. Electroosmosis is a well known effect in the capillary electrophoresis of the macromolecules [23]–[25], where electroosmotic velocity is usually several-fold higher than electrophoretic velocity of the medium size proteins. Electroosmosis is used in practice, e.g. for pumping fluids [26] and mixing reactants in microfluidics [27].

The detailed theoretical study of electroosmosis in the capillary tube both with open and closed ends was presented in [28]. It showed that the external electric field caused the circulating electroosmotic flow in the cylinder with the closed ends. The flow of fluid in this closed volume is in the direction of electric field in the vicinity of cylinder wall and in the opposite direction in the vicinity of the cylinder axis; the total flow of fluid through any cross section of this volume is obviously zero. The necessary and sufficient conditions of the electroosmotic circulation of the fluid are the presence of the surface with electric double layer and of the component of electric field parallel to this surface. These conditions are simple and are likely to be satisfied in many biological systems. Surprisingly, intracellular electroosmosis has been ignored so far with the exception of one scientific field–plant physiology, where the series of studies [29]–[35] published mostly in 1950–1970s discussed the possible role of electroosmosis in the transport of water and nutrients in the plant tissues. In particular, electroosmosis was hypothesized to be the mechanism for the transport of sugars along the cytoplasm of the sieve tube cells of the phloem of the higher plants, while the electric potential was assumed to be generated by the active uptake of the K^+^ ions by the companion cells of the phloem [30]. Similar hypothesis was formulated in [31]–[32], with the exception that electric potential was assumed to be generated by the diffusion of H^+^ ions. The electroosmotic flow of water was measured directly in a live *Nitella translucens* cell (1 cm×0.1 cm cylindrical cell) by applying external electric current (1.2 µA, 0.05 V) [33]. Similar experiments reported on the electroosmotic flow in the vascular strand of the water plant *Nymphoides peltatum* [34], and later in the mesocotyls of maize seedlings [35]. The observed electroosmotic flow was quite substantial (estimated at 100–120 molecules of water per ion of the external electric current) and was assumed to pass from one cell to another through the porous sieve plates. However, these experiments remained restricted to the plant physiology, likely due to the difficulties of the similar measurements in the smaller animal cells. Now, that the presence of cytoplasmic electric fields is proved by direct measurements in the living astrocytes [9], it is time to quantitatively evaluate the potential role of electroosmotic transport in any polarized cell. Almost all cell types are polarized, i.e. have some degree of electrical asymmetry. The classical examples of the polarized cells are epithelial cells, neurons, and embryonic cells.

Recently, we have developed and presented at the COMSOL users conference the simplistic two-dimensional minimal model of the intracellular electroosmotic flow in the square-shaped cell showing the potential role of electroosmosis in the intracellular transport of messenger proteins to the electrically neutral nucleus [36]. Here, we present two more detailed and realistic models (the first model is axisymmetric and the second is true three-dimensional) with the material parameters estimated and justified by the extensive literature search reviewed in the “Methods S1. Parameters Justification”. Unlike the model of [36] which assumed electrically neutral nucleus and simulated the transport of large proteins (diffusion coefficient D = 10^−12^ m^2^/s), the new models consider negatively charged nucleus and small signaling proteins (molecular weight∼ 45 kDa, D = 14·10^−12^ m^2^/s). In addition, the more accurate boundary and initial conditions were used in the new models. Importantly, both old and new models predict substantial role of electroosmosis in the intracellular transport. Methods section contains the detailed description of the assumptions and the equations of the models.

Briefly, in the first model, we simulate the transport of the negatively charged messenger proteins towards the negatively charged nucleus of the polarized cells. We compare the speed and efficiency of the transport with and without the electroosmotic flow and demonstrate that electroosmotic flow can facilitate the transport of negatively charged messenger proteins to the negatively charged nucleus. We also suggest that electroosmotic flow can influence the motion of proteins during the fluorescence recovery after photobleaching (FRAP) experiments and therefore might contribute to the values of apparent diffusion coefficients measured in these experiments. In the second model, we simulated FRAP experiments with and without electroosmosis and demonstrated that the presence of electroosmosis made the apparent diffusion coefficient dependent on the position of the bleaching spot, which might explain variability in the results of the FRAP experiments. The goal of these models is to present the quantitative analysis of the simplest cellular configuration where the effect of the intracellular electroosmotic flow is substantial. Real cells contain numerous charged surfaces and numerous electrical double layers; therefore it is most likely that electroosmotic flow effect is even more important in the real cells than in our idealized case. One can envision the existence of the complicated pattern of the intracellular electroosmotic flow along the charged surface of endoplasmic reticulum, cytoskeleton, and other organelles. Finally, we discuss the possible broader implications of electroosmosis in the in vivo transport of biomolecules in the context of the papers demonstrating cytoplasmic circulation in plant cells, embryonic cells, and neurons.

## Methods

### Model Description

Following the schematic presentation of [4], we modeled the polarized cell as a cylinder with a spherical nucleus in the center. Similarly following [4], the ion pump/channel activity was assumed asymmetrically distributed: electric current was entering from the upper horizontal side of the cylinder and leaving the cell through the opposite side of the cylinder. We intentionally started with this simple configuration in order to show that electroosmotic circulation inside the cell did not require complicated conditions, but could occur in any configuration where the ideal symmetry is broken. Obviously, the presence of the organelles with the charged membranes (e.g. mitochondria, and endoplasmic reticulum) in the real cell would break the symmetry and create the component of the electric field parallel to the interfaces even in the spherical cell. However, these more complicated cases are computationally intense and will be the topic of the future studies.

The parameters of the model used in the simulation are presented in Table 1. The mathematical description of the model is presented below. The justification of the choice of the parameters based on the review of literature data together with some computational details are presented in the (“Methods S1. Parameters Justification”). Simulations were performed with the COMSOL 4.2a Multiphysics software.

#### Electric Field

Electric properties of the cytoplasm and nucleus were taken from the experimental study [37] as σ_1_ = 0.25 S/m, ε_1_ = 60; σ_2_ = 0.5 S/m, ε_2_ = 120, where σ and ε are conductivity and relative dielectric permittivity. The electric charge relaxation time is determined by 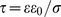 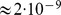
s, where 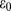-dielectric permittivity of vacuum. The timescale of the intracellular biological processes is much longer, i.e. 10^−6^ s
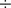
100 s [38], therefore Maxwell's equations of electromagnetism can be reduced to the equations of the continuity of electric current. In this model, we assumed the absence of the external sources of the electric current, except at the top and bottom walls of the cylinder, where current with equal density enters and leaves the cell (representing the ionic flux that the cell “drives through itself” [4]). Therefore electrical part of the model is reduced to the following simple equations:



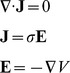
(1)


with the boundary conditions of equal inward and outward current densities at the top and bottom of the cylinder:



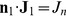
(2)


and conditions of the fixed potential and electric current continuity at the interface of cytoplasm and nucleus:



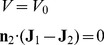
(3)


where **J**, **E**–vectors of electric current density and electric field strength, *V*–electric field potential, **n**–normal to the boundaries and interface, *V_0_* = −0.033 V–potential of the nucleus, following [17].

#### Electroosmotic Flow

In general, electroosmotic flow (similar to any other fluid flow) should be described by the Navier-Stokes equations. However, these equations can be dramatically simplified, since cytoplasm (similar to water) is practically incompressible and since the Reynolds number for the flow of the intracellular fluid is extremely small: 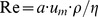
≤10^−3^ (given the cell size 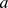
is about 10^−5^ m, dynamic viscosity of cytoplasm is η = 0.002 Pa·s, [39], density is about the same as of water ρ = 10^3^ kg/m^3^, and maximum velocity 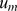 is below 10^−4^ m/s). For such low Reynolds number, the fluid flow is described by the Stokes equations:



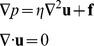
(4)


where 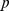
- pressure, 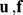-vectors of fluid velocity and applied external force density. Paper [28] provided the analytical solution of the equation (4) for the infinitely long cylinder, where the external force 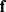 was determined as a product of external electric field and the electric charge of the double-layer at the solid-fluid interface. When the diameter of the cylinder is much larger than the double-layer thickness 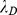 (about 1 nm in cytoplasm), the exact analytical solution is asymptotically equal to the solution obtained when all electric charge and hence external force are concentrated at the boundary, therefore resulting in the Helmholtz-Smoluchowski slip boundary condition at the solid-fluid interface:



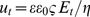
(5)


where 
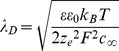
, 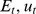-tangential components of electric field and fluid flow velocity, *k_B_* -Boltzmann constant, *T*-temperature, *F*- Faraday number, *z_e_*–valence number of the ions forming the double-layer, and 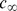
- ion's molar concentration in the bulk solution. The above electroosmotic slip boundary condition (5) was further justified for more complicated geometries in [40]–[42] and used in modeling and design of electroosmotic pumps and mixers [26], [42]. We used the Helmholtz-Smoluchowsi boundary condition (5) on the cylindrical and filleted part (introduced to avoid nonrealistic singularities in the corners) of the cellular membrane model, i.e. everywhere on the cellular membrane except the top and bottom walls.

The top and bottom walls are considered permeable to the electric current, i.e. to the flow of small ions through the open ion channels, therefore no electric double-layer is present on these surfaces. Similarly, the nuclear envelope is known to be permeable to small ions and molecules [2], meaning that no electric double-layer exists at the boundary of the nucleus as well. The choice of the boundary condition at the above surfaces is not obvious, since both membranes are viscoelastic and deformable. We chose the slip boundary condition on these surfaces formulated as:



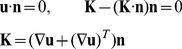
(6)expressing that there is no flow across the boundary and no viscous stress in the tangential direction. The justification of this choice of boundary conditions for biological membranes is presented in the (“Methods S1. Parameters Justification” file).

#### Transport of Macromolecules

In this study, we developed and analyzed two models of intracellular transport of proteins. In the first model, we studied the transport of negatively charged messenger proteins from the cellular membrane to the nucleus. In the second model, we examined the possible role of electroosmosis in the experiments on fluorescence recovery after photobleaching. In both models we assumed the concentration of proteins of interest being much lower than the concentrations of other major proteins in the cytoplasm and therefore we ignored nonlinear concentration effects, i.e. the interaction/competition between the proteins of the same kind. Interactions of the proteins of interest with other proteins, e.g. binding sites of the cytoskeleton, were introduced through using the apparent diffusion coefficients from the FRAP experiments.

**The first model (transport of messenger proteins to the nucleus)** is described by the following diffusion-convection equation:



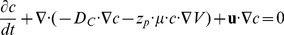
(7)with the “no flux” boundary condition everywhere at the cellular membrane



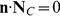
(8)


where 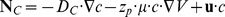,

*c*- concentration of the messenger protein, *D_C_*–apparent diffusion coefficient of the messenger protein in the cytoplasm, and 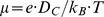 -mobility of the single charged protein, *e*-electron charge, *z_p_*–apparent valence number of the protein, 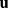-vector of fluid velocity.

The transport of the messenger protein inside the nucleus was assumed purely diffusional, since both electric field and electroosmotic flow were absent inside the nucleus according to the solutions of the above electric field and hydrodynamic problems.



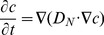
(9)with the continuity of fluxes at the interface of cytoplasm and nucleus:



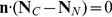
(10)


where 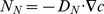, *D_N_* – diffusion coefficient of the messenger protein in the nucleus, which we assumed much smaller than *D_C._*

The initial distributions of messenger protein molecules were modeled as narrow zones near the cylindrical wall:



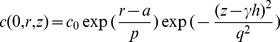
(11)


where 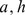-radius and height of the cell, *p* = 2•10^−8^ m and *q* = 10^−8^ m determines the depth and the width of the initial distributions, 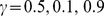
- describe the position of the center of the distribution at the center, upper part and lower part of the cylindrical surface. Similarly, the initial distributions at the top and at the bottom surfaces were described by:
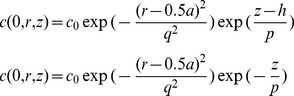
(12)


#### The second model (FRAP experiment)

To estimate the possible impact of electroosmotic flow on the measurements of the diffusion coefficients in the fluorescence recovery after photobleaching experiments, we developed the 3D model of the cell similar to the above axisymmetric model. The cell was modeled as a cylinder. The initial condition for the concentration of the fluorescent protein, resulting from bleaching by laser beam was modeled by eq. (13), following [43]–[44].



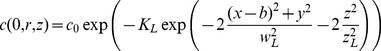
(13)


where 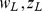 are the laser beam radii in the radial direction 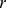 and in the axial direction 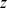, and 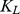
is bleach efficiency.

**Figure 1:**
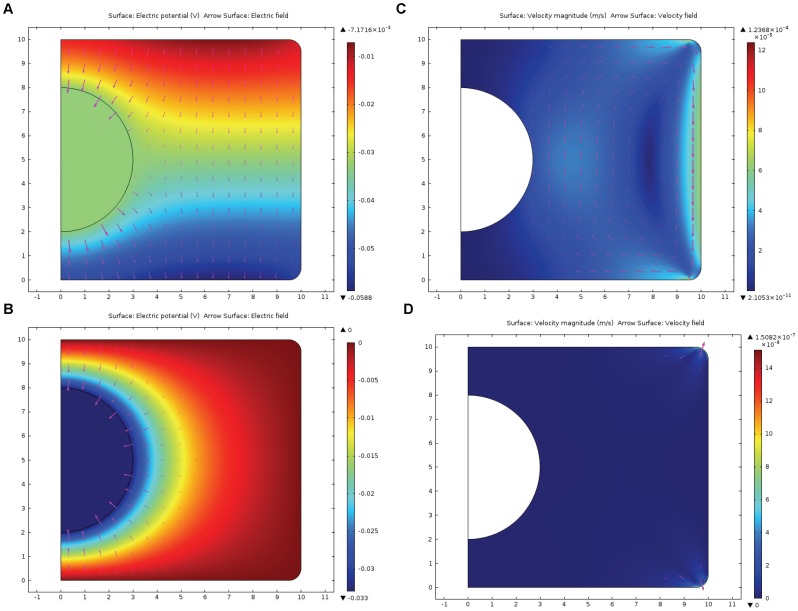
Electric field and electroosmotic velocity in the cylindrical model of the polarized and non-polarized cells. Results of the computer simulation of electric potential, electric field and electroosmotic flow velocity in the axisymmetric model of the cell (cylinder of 10 µm radius and 10 µm height, nucleus–sphere of 3 µm radius, located in the center of the cylinder). Mathematical model is described by eqs. (1–6); parameters are presented in Table 1. Figure 1A represents electric potential (color map) and electric field strength (arrows) in the model of the polarized cell; inward current enters through the top of the cylinder and leaves the cell through the bottom of the cylinder (eq. 2). Electric potential is in the range −0.007÷−0.059 V; strong component of electric field parallel to the cylindrical surface is present. For comparison, Figure 1B presents electric potential and field in the non-polarized cell (zero inward and outward current); here the component of electric field parallel to the surface is practically absent. Figure 1C demonstrates electroosmotic flow circulation (color represents magnitude of the velocity, arrows represent direction) caused by electric field distribution in the polarized cell (Fig. 1A), maximum value of fluid velocity 120 µm/s is in the vicinity of the cylindrical wall, while the average magnitude of the fluid velocity across the cytoplasm is 20 µm/s. Fluid circulates, moving downwards near the cylindrical wall and upwards near the nucleus. For comparison, Figure 1D demonstrates that electroosmotic flow is practically absent (except the filleted corners) in the model of a non-polarized cylindrical cell, where electric field parallel to the surface is absent.

The switch from the axisymmetric to the true 3D model was required since the bleaching laser beam in the FRAP experiments was not necessary focused at the center of the cell surface. As seen in the first model, electroosmotic velocity was higher at the regions closer to the cylindrical wall, so it was reasonable to assume that the impact of the electroosmotic flow would be stronger for the larger bias *b* of the cylindrical laser beam relative to the axis of the cylindrical cell. On the other hand, as seen in the results of the first model (Fig. 1C), the presence of the nucleus located at the center of the cell did not cause large disturbance to the electroosmotic flow profile. Therefore, and in order to enable computationally feasible simulation, we ignored the presence of nucleus in this model. Other than that we used the same assumptions and the same equations as in the axisymmetric model above.

### Implementation of the Models in COMSOL 4.2a

The above models of messenger protein transport and FRAP experiments in the polarized cells were developed within COMSOL 4.2a Multiphysics (COMSOL, Inc, Burlington, MA, USA) software environment, which implemented the finite element approach to the solution of the sets of partial differential equations. In addition to the core COMSOL, the software package included MEMS module and Microfluidics module. Our model took the advantage of the following predefined templates-“physics interfaces”: Electric Currents, Creeping Flow, and Transport of Diluted Species; the first two in the stationary and the last one in the time dependent mode. The default–physics controlled meshing was used with the option the “finest” or the second “finest” mesh selected to ensure proper conversion and precision of the calculations. Mesh in the axisymmetric model contained: 12480 triangular elements, 160 quadrilateral elements, 434 edge elements, and 12 vertex elements. Mesh in the 3D model contained: 111900 tetrahedral elements, 5424 prism elements, 9126 triangular elements, 240 quadrilateral elements, 366 edge elements, and 17 vortex elements. Computations were performed with the iMAC computer (2 GHz Intel Core 2 Duo; 4 GB, 667 MHz DDR2 SDRAM; Mac OS X 10.5.8). Typical duration of the computation for the given initial conditions was about 30 minutes. The model files (.mph) which can be executed within COMSOL 4.2a or higher are deposited at ModelDB and are freely available for download: http://senselab.med.yale.edu/ModelDB/ShowModel.asp?model=147740.

## Results

### Transport of Messenger Proteins to the Nucleus

Here we present the results of the axisymmetric model of the transport of negatively charged (*z_p_* = −2) messenger proteins in the polarized cell, which is modeled as a cylinder (10 µm radius, and 10 µm height). As justified in the Introduction and in the (“Methods S1. Parameters Justification” file), the polarized cell was pumping electrical current through itself [4], which was entering through the top and leaving through the bottom of the cylinder. The cell nucleus was considered negatively charged (potential *V_0_* = −0.033 V). Figure 1 presents the results of the solution of the electric (eq.1–3) and hydrodynamic (eq. 4–6) parts of the model. Fig. 1A presents the electric potential distribution that ranges from −0.007 V to −0.059 V, while the arrows present the direction and relative strength of electric field. For comparison, Fig. 1B presents electric field in the non-polarized cell, where the cellular membrane is assumed equipotential and the nucleus is negatively charged relatively to the cytoplasm. Importantly, there is a strong component of electric field parallel to the cylindrical surface of the cellular membrane in case of the polarized cell, which is necessary to generate the electroosmotic flow. Figure 1C demonstrates electroosmotic flow circulation caused by electric field distribution in the polarized cell (Fig. 1A), the zeta-potential of the cylindrical surface of the membrane is assumed ζ = −0.05 V (see “S1 Methods. Parameters Justification” file). The maximum value of fluid velocity *v_osm max_* = 120 µm/s is in the vicinity of the cylindrical wall, while the average magnitude of the fluid velocity across the cytoplasm is *v_osm av_* = 20 µm/s. Cytoplasm fluid circulates, moving downwards near the cylindrical wall and upwards near the nucleus. For comparison, Figure 1D demonstrates that electroosmotic flow is practically absent in the model of non-polarized cylindrical cell (average velocity across the cell *v_osm av_* = 0.001 µm/s, maximum velocity *v_osm max_ = *0.15 µm/s, which is present only in the corners of the cell).

**Figure 2:**
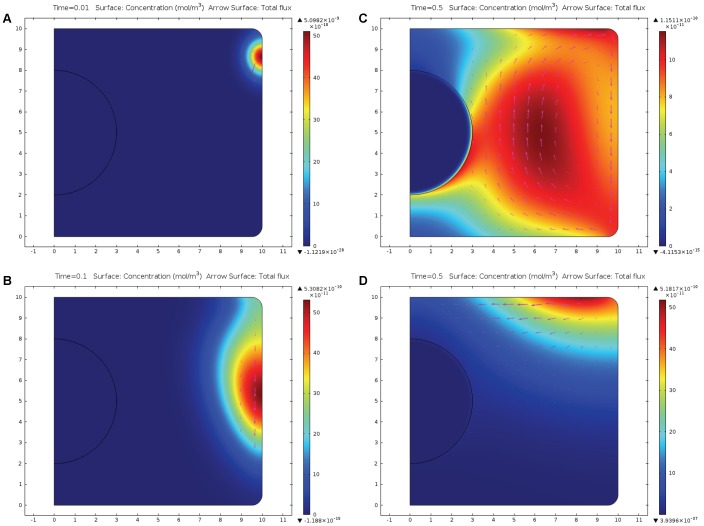
Evolution of the concentration of messenger proteins initially positioned near the upper corner of the cell. Results of the computer simulation of the evolution of concentration (color map) and flux (arrows) of the negatively charged messenger proteins (charge = −2e) initially located near the upper corner of the cell. Mathematical model is described by eqs. (7–11). Figure 2A presents the distribution of the messenger proteins 0.01 s after initiation. At t = 0.1 s, as seen at Fig. 2 B, proteins drift with the electroosmotic flow along the cylindrical surface, while at t = 0.5 (Fig. 2C) they are carried by the electroosmotic flow towards the nucleus. The strong flux of messenger proteins to the surface of the nucleus is formed. For comparison, Fig. 2 D demonstrates what happens with messenger proteins in case of the absence of electroosmotic flow, e.g. when zeta-potential, ζ = 0. In this case, negatively charged proteins migrate in the electric field to the least negative part of the cytoplasm, i.e. upper corner of the cell, from where only the small fraction of messengers can reach the nucleus by diffusion against the electric field.

Figure 2 represents the evolution of the distribution of concentration and flux of the messenger proteins that were initially positioned near the upper corner of the cylindrical surface. At t = 0.1 s, as seen at Fig. 2B, proteins drift with the electroosmotic flow along the cylindrical surface, while at t = 0.5 (Fig. 2C) they are carried by the electroosmotic flow towards the nucleus. Together with diffusion, it forms a strong flux of messenger proteins to the surface of the nucleus. For the sake of comparison, Figure 2D demonstrates what happens with messenger proteins in case of the absence of electroosmotic flow, e.g. when zeta-potential, ζ = 0. In this case, negatively charged proteins (*z_p_* = −2) migrate in the electric field to the less negative part of the cytoplasm, i.e. upper corner of the cell, from where only the small fraction of messengers can reach the nucleus by diffusion against the electric field.

**Figure 3:**
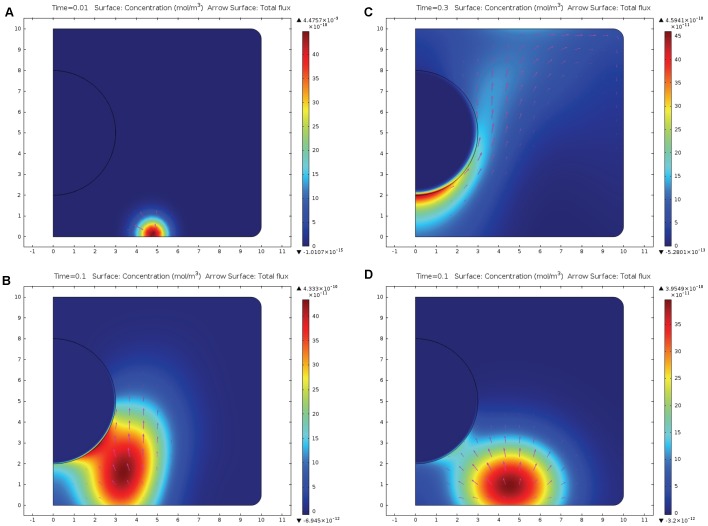
Evolution of the concentration of messenger proteins initially positioned at the bottom of the cell. Mathematical model is the same as in Figure 2 except the initial condition that is determined by eq. (12). Figure 3A presents the distribution of the messenger proteins 0.01 s after initiation. Figure 3B demonstrates that at t = 0.1 s a lot of messenger proteins reach the nucleus due to both electroosmotic flow and migration caused by the electric field. Figure 3C illustrates that at t = 0.3 s the layer of messenger proteins is formed in the vicinity of the nucleus. Figure 3D (ζ = 0) demonstrates that the transport is much slower by electromigration relative to electromigration combined with electroosmosis (Fig. 3B).

Figure 3 illustrates the case where the messenger proteins are initially positioned at the bottom of the cell and are carried to the nucleus both by electroosmotic flow and due to migration in the electric field. Comparison of Fig. 3. 3B and 3D shows however, that at t = 0.1 much more messengers are able to get to the nucleus when electroosmotic flow is present (Fig. 3B) than in the case when electroosmotic flow is absent (ζ = 0) shown in Fig. 3D. At t = 0.3 s, a layer of messenger proteins is formed in the vicinity of the nucleus surface; similar concentration distributions are established at this time point both in case where electroosmosis is present (Figure 3C) and where it is absent (data not shown). Importantly, however, messengers get to the nucleus much faster when the electroosmosis is involved (compare Fig. 3B and 3D).

**Figure 4:**
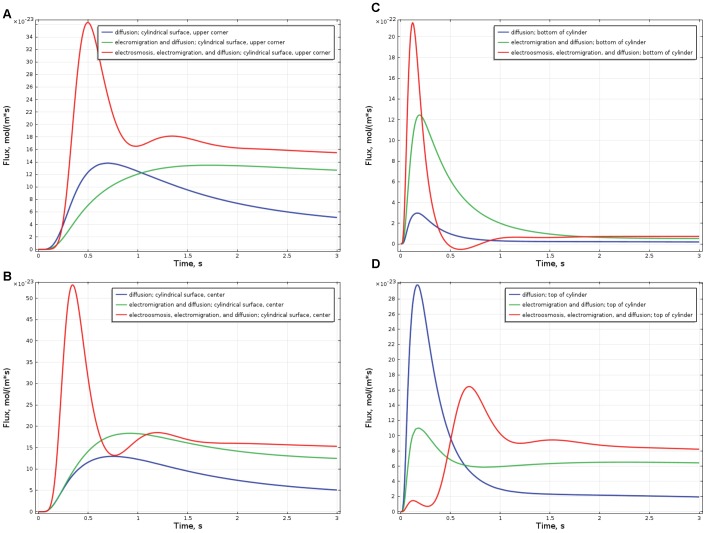
Time dependence of the flux of the messenger proteins onto the nucleus. The flux of the messenger proteins normal to the surface of the nucleus is integrated over this surface. Red curves present the total flux due to electroosmosis, electromigration, and diffusion. For comparison, blue curves present the flux due to pure diffusion, and green curves present the flux due to diffusion and electromigration. In Figure 4A messengers are initially located near the upper corner of the cell (as in Fig. 2), in Figure 4B near the center of the cylindrical surface, in Figure 4C at the bottom of the cylinder (as in Fig. 3), and in Figure 4D at the top of the cylinder. Note that everywhere, except Fig. 4D, electroosmosis facilitates faster and more intense transport of messengers onto the surface of the nucleus. See text for more detailed discussion.

Figure 4 demonstrates the time dependencies of the flux of the messenger proteins on the surface of the nucleus integrated along this surface. For the sake of comparison, we present the flux caused by electroosmosis, electromigration and diffusion-red line, flux due to electromigration and diffusion in case where electroosmosis is absent (ζ = 0)–green line, and flux due to diffusion only, which would occurs if both ζ = 0 and *z_p_* = 0–blue line. Figure 4A illustrates the case where messenger proteins are introduced near the upper corner of the cell; the maximum flux is established at t = 0.5 s and the flux “with electroosmosis” is more than two-fold higher than due to pure diffusion, or diffusion and electromigration. Figure 4B illustrates the flux of the messengers initially located at the center of cylindrical surface; the maximum flux is attained at about t = 0.3 s and is 3–4-fold higher “with electroosmosis” than due to pure diffusion and due to diffusion with electromigration. Figure 4C presents the case where messenger proteins are located initially at the bottom of the cylinder (as in Fig. 3). The maximum flux is established at t ≈ 0.1 s, and the flux “with electroosmosis” is more than six-fold higher than due to pure diffusion and two-fold higher than due to diffusion and electromigration.

The initial position of the messenger proteins along the circulating pattern of the electroosmotic flow (shown in Fig. 1C) determines both the intensity of the flux and the time when the maximum of the flux is attained. Bottom of the cylinder (Fig. 4C) is quite close upstream on the electroosmotic flow circulation from the nucleus, therefore the transport of the messenger to the nucleus is fast: t_max_≈0.1 s, F_max_≈22·10^−22^ mol/(m·s). The center of the cylindrical surface (Fig. 4B) is further upstream and therefore the flux maximum is lower and is attained later: t_max_≈0.3 s, F_max_≈5.4·10^−22^ mol/(m·s). The upper corner of the cylindrical surface is even further upstream (Fig. 4A), which results in even later arrival: t_max_≈0.5 s and lower maximum F_max_≈3.6·10^−22^ mol/(m·;s). It is of interest to compare the cases, where the messengers are initially located at the bottom of the cylinder, r = 5 µm (Fig. 3 and Fig. 4C) and symmetrically at the top of the cylinder r = 5 µm (Fig. 4D). These locations are at the equal distances from the cell nucleus and therefore the transport due to pure diffusion is the same from both locations (see blue lines at Fig. 4C and 4D, t_max_≈0.2 s, F_max_≈3.0·10^−22^ mol/(m·s)), however, in the first case electromigration and electroosmosis facilitate transport (red line, Fig. 4C, t_max_≈0.1 s, F_max_≈22·10^−22^ mol/(m•s)), while in the second case both of the factors force the messengers to move away from the nucleus along the circulation loop of the electroosmotic flow and therefore the flux maximum is much lower and the arrival time is much later (red line, Fig. 4D, t_max_≈0.7 s, F_max_≈1.6·10^−22^ mol/(m·s)).

As shown above, the presence of electroosmotic flow causes the several fold change of the messenger protein flux intensity and the protein arrival time. For the larger part of the cellular membrane surface, the presence of electroosmotic flow intensifies the transport of the messengers and results in the higher and narrower peaks of the flux on the nuclear membrane which is preferential for the optimal flow of information in cell signaling, as discussed in [12]–[13]. The arrival time and the shape of the flux peak depend on the initial location of the messengers and therefore can convey the information of the location of the ligand binding at the cellular membrane, which might be important for the cell and is lost in case of diffusional transport as argued in [13]. The presence of the electroosmotic flow explains the fast transport of negatively charged messenger proteins to the negatively charged nucleus which would be otherwise quite slow for the messengers starting from the larger part of the cellular membrane. Another attractive feature of electroosmotic transport is its circulatory pattern which provides the mechanism for fast “recycling” of the extra unused messenger proteins from the nucleus back to the cellular membrane, where it can be used for transmission of the new messages.

### Possible Influence of Electroosmotic Flow on the Measurements of Diffusion Coefficient in the FRAP Experiments

Fluorescence recovery after photobleaching is a widely used tool for estimating diffusion coefficients of the fluorescently labeled molecules in cells [43]–[46]. The early models of FRAP assumed the infinite homogeneous medium and considered bleaching as a first-order linear process, taking negligible time compared to diffusion. Based on these models, an equation for fluorescence evolution was established, enabling the estimation of diffusion coefficients from the time 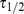
required for the recovery of the 50% of fluorescence level in the bleached spot [45]. The later model [44] argued that for the fast diffusing molecules, the significant fluorescence recovery could occur by the time the first photobleaching image was acquired, and therefore diffusion during the bleaching process needed to be modeled and the diffusion coefficients determined in FRAP experiments needed to be corrected. The goal of our below study is to show that the presence of electroosmosis might cause even larger errors in determination of the diffusion coefficients from FRAP experiments. The bleaching profile used as the initial condition for the fluorescent protein concentration is described by eq. (13), parameters of the profile are acquired from [44] and presented in Table 1.

**Figure 5:**
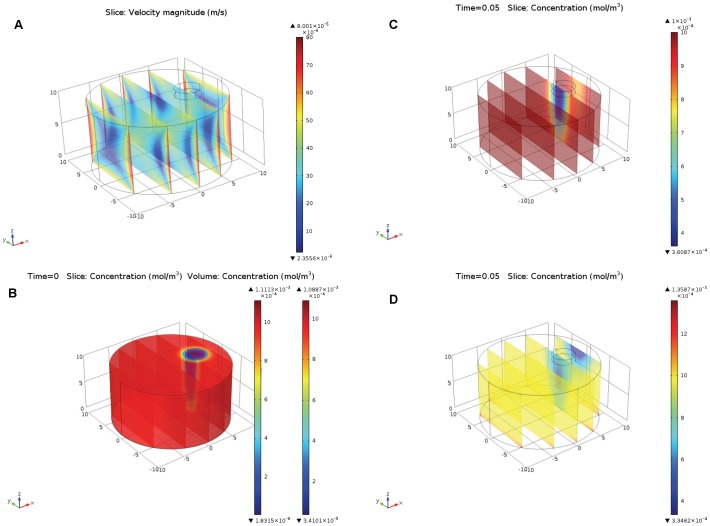
The 3D model of the fluorescence recovery after photobleaching in the polarized cell. Mathematical model described by eqs. (7–8) with initial condition (13), representing photobleaching by laser beam. Small cylinder near the top of the cell represents the area across which the recovery of photobleaching is observed (averaged). Figure 5A represents the magnitude of electroosmotic flow velocity (similar to Fig. 1C, but in 3D). Figure 5B presents the concentration of the fluorescently labeled protein after photobleaching, t = 0. Figure 5C–recovery of the fluorescence (concentration of the fluorescent protein) due to pure diffusion, t = 0.05. Figure 5D–recovery of the fluorescence due to electroosmosis and diffusion.

Figure 5 illustrates the results of the modeling: Fig. 5A–electroosmotic flow velocity, Fig. 5B-depletion of the fluorescent labeled protein after photobleaching t = 0, Fig. 5C- recovery of the fluorescence after photobleaching at t = 0.05 s in case where only diffusion is present (ζ = 0, *z_p_* = 0), Fig. 5D–recovery after photobleaching at t = 0.05 s where both diffusion and electroosmosis are present (ζ = −0.05 V). The small cylinder near the top of the cell represents the area across which the concentration (fluorescence) is observed and averaged for determination of the 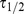
value.

**Figure 6:**
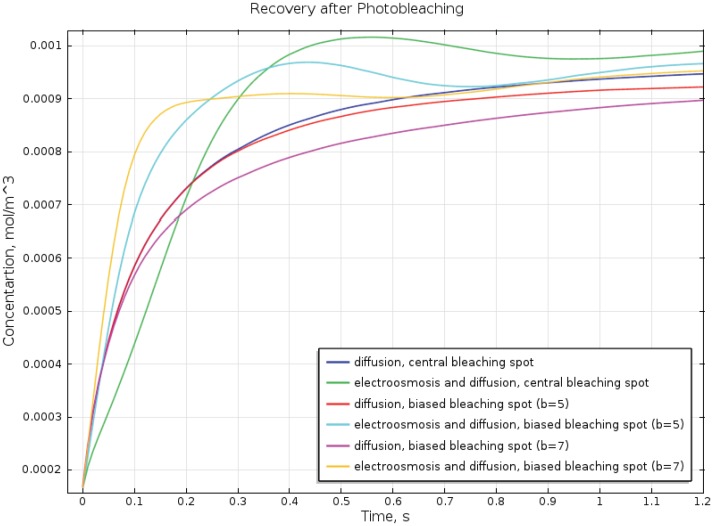
Dynamics of the recovery of the fluorescence after photobleaching. Concentration of the fluorescently labeled protein averaged over the small cylinder near the top of the cell presented in Fig. 5. Blue, red, and magenta curves represent recovery of the fluorescence due to pure diffusion for central (blue), biased b = 5 µm (red), and biased b = 7 µm (magenta) locations of the bleaching spot. In case of pure diffusion the time of 50% recovery does not depend on the position of the bleaching spot. On the contrary, in case of electroosmosis and diffusion, the recovery curves differ substantially for the different locations of the bleach spot: central-green, b = 5 µm -cyan, b = 7 µm-yellow. The 50% recovery time differs for these locations: 0.12 s, 0.058 s, and 0.04 s. Therefore diffusion coefficients determined from these values of recovery time might differ 3-fold as well. See text for more detailed discussion.

Figure 6 demonstrates the dynamics of the recovery of fluorescence which is represented by the concentration of fluorescent labeled protein averaged across the small cylinder. The presented curves differ by the position of the bleaching spot (*b* = 0, 5 µm, 7 µm) and by the values of the zeta potential: ζ = 0–diffusion only, ζ = −0.05 V–diffusion and electroosmosis. In case of central bleaching spot, the vertical component of the electroosmotic flow makes the fluorescence recovery slower than it would be due to diffusion only (compare dark blue and green curves), since the axial electroosmotic flow moves the bleached protein molecules from the depth of the bleach spot to the cell surface and counteract the diffusion of non-bleached molecules from the periphery to the axis. The value of 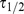
in case of central bleach spot is about 0.07 s due to pure diffusion and is about 0.12 s when electroosmosis is present. Later, however, the overall better mixing due to electroosmosis results in the higher concentration of the fluorescently labeled protein in the bleached spot (the green curve is above dark blue at t>0.22 s). In case of eccentric position of bleaching point (*b*≠0), the substantial horizontal component of the electroosmotic flow results in the better mixing from the very start of the recovery. The pure diffusion curves for *b* = 0, 5 µm, 7 µm have the common initial part and all cross the 50% recovery line (*c* = 0.0005 mol/m^3^) at 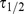 = 0.07 s, while the electroosmosis reduces the 50% recovery time to 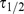 = 0.058 s for b = 5 µm and to 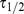 = 0.04 s for b = 7 µm. Thus, in our model, the presence of electroosmotic flow results in the up to 3-fold differences in the values of 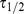 depending on the position of the bleaching spot relative to the center of the cell. According to [45], the diffusion coefficient estimated from the FRAP experiments is proportional to 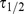 and therefore would differ 3-fold as well.

The dependence of the apparent diffusion coefficient on the positioning of the bleaching spot can be one of the reasons explaining the large run-to-run variability in diffusion coefficients determined from FRAP measurements, as for example in [47] where the apparent diffusion coefficient for the green fluorescent protein varied from 1.2 µm^2^/s to 94.2 µm^2^/s (mean = 17.3 µm^2^/s; std = 31.1 µm^2^/s), or [48] where the apparent diffusion coefficient of yellow fluorescent protein was estimated at 20.6±5.0 µm^2^/s.

## Discussion

Above, we demonstrated that electroosmotic flow could substantially influence the intracellular transport of the biomolecules in the polarized cells. As an illustration of this influence we modeled the transport of negatively charged phosphorylated molecules of messenger proteins to the negatively charged nucleus and demonstrated that the flux of the messengers on the nucleus could be up to 4-fold higher in the presence of electroosmosis than due to pure diffusion or due to diffusion and migration in electric field. Similarly, we demonstrated that the presence of electroosmosis could influence the values of the time of the 50% recovery of the fluorescence in the FRAP experiments and therefore the values of apparent diffusion coefficients estimated from these experiments. Importantly, the recovery time was shown to differ up to 3-fold depending on the positioning of the bleach spot relative to the cellular membrane which supported electroosmotic flow. Here again, we need to emphasize that the structure of the real cell is much more complicated than our models and includes charged surfaces of multiple organelles that are able of supporting the electroosmotic flows of their own. Positioning of the bleaching point relative to these charged surfaces might influence the fluorescence recovery time as well. Our goal was to present the simplest configuration where this influence could occurr.

In the above calculations, we used the parameter values determined in the extensive literature review presented in the “S1 Methods. Parameters Justification” file. As seen in the reviewed papers, the range of the possible parameter values is quite broad. It is of interest to estimate how different choice of parameter values could influence the results of our study. The relative role of diffusion and electroosmosis can be roughly estimated by comparing the time required to travel across the cell by diffusion and by electroosmosis: 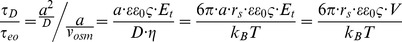
(14)


where viscosity *η* is canceled out when *D* is substituted by the Stokes-Einstein equation (s1) (see “S1 Methods. Parameters Justification” file). Importantly, it means that the relative role of electroosmosis is proportional to 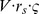
- the product of the electric potential difference across the cell, Stokes radius of the biomolecule, and the zeta-potential of the cellular membranes. The values used in our model (*V*≈0.05 V, ζ = −0.05 V, 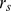 = 3.1 nm–MW = 45 kDa) resulted in about 3–4-fold higher flux due to electroosmosis relative to pure diffusion.

It is of interest to look at the possible broader implications of electroosmotic flow in the biological phenomena. One can expect proportionally higher relative role of electroosmosis in the transport of larger proteins and viruses which have much larger Stokes radii, e.g. 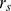 = 10.7 nm for human fibrinogen MW = 387 kDa [49], 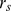 = 28 nm, HIV-1 virus [50]. Viruses and vesicles are known to be transported actively, i.e. by motor proteins. The active microtubule dependent velocity of the typical virus movement is about 1–3 µm/s [51]–[53]. As described in [51], “virus particles not only followed single microtubules but also hopped from one to another during their egress”. Similar, saltatory movement was observed for microtubular based transport of other cellular cargos [51]. It is likely, that at least during these “hopping stages”, when viruses or other cargoes are not bound to the microtubules, they move together with the cytoplasm in which they are immersed. As estimated above (see Fig. 1B), the average electroosmotic velocity across the cell in our model was 20 µm/s, i.e. about 10-fold higher than the typical velocity of the active transport of viruses in the cytoplasm. Therefore, it is quite likely that the active transport by motor proteins of viruses, vesicles and large proteins is influenced/complemented by the electroosmotic flow in the polarized cells. Various transport mechanisms are likely closely intertwined similar to the described in [54]–[55], where the active motor-driven transport along the cytoskeletal filament networks causes fluid advection which in turn causes the transport of unbound proteins. It is quite possible (although not discussed in these papers) that the surface of the filaments is charged and therefore supports electroosmotic flow which further complicates the interaction between advection and active transport.

Several papers discuss cytoplasmic circulation which was observed in the large plant and fungal cells [56]–[62] where the directed flow of cytosol and organelles was estimated at the velocities about 80–100 µm/s. The most popular explanation of the mechanism of the cytoplasmic streaming is that “the directed motion of myosin motors on the actin bundle tracks, located in the cellular wall can somehow entrain the cytoplasmic fluid, and that this in turn sets the vacuole into motion by transferring momentum through the fluid membrane separating it from the cytoplasm” [59]. The quantitative representation of this hypothesis is the sliding wall theory [58], [60] which results in the flow pattern that was confirmed by the recent direct nuclear magnetic resonance velocimetry experiments of flow inside the vacuole of single internodal cells [61]. The mechanism of momentum transfer from myosin to endoplasm and vacuole is not trivial and was theoretically examined in [62], where it was shown that individual myosin molecules running on the actin tracks are by themselves ineffective in setting the cytosol or the vacuole into motion. To entrain such motion, the authors of [62] envisaged the existence of an elastic network, or gel, incorporating the moving motors and extending into the endoplasm.

It is of note that electroosmotic flow profile is also quantitatively described by the sliding wall boundary condition, i.e. Helmholtz-Smoluchowski slip boundary condition at the solid-fluid interface eq. (5). The range of the electroosmotic flow fluid velocities demonstrated in our model (20÷120 µm/s, see Fig. 1B) is also comparable with the velocities of cytoplasmic streaming in the plant cells, therefore it seems probable that electroosmosis might play a role in the mechanism of the cytoplasmic streaming. The likelihood of the involvement of electroosmosis in the above phenomena is supported by the experimental finding that cytoplasmic streaming can be inhibited by the generation of action potential caused by the activation of Ca^2+^ and Cl^−^ channels [63]–[64], meaning that electrical dimension is involved in the mechanism of the cytoplasmic streaming.

The cytoplasmic streaming was experimentally observed not only in the plant cells but in the developing cells of c.elegans embryo [65], fertilized mouse egg [66], and during the axon formation in the hippocampal neurons [67]. The cytoplasmic circulation with the flow velocity of 0.17 µm/s was observed in the one-cell stage embryo of c.elegans and was regarded as hydrodynamic motion of the cytoplasm driven by actin-myosin on a thin layer near the cell cortex [65]. The rhythmical cytoplasmic movements (mean velocity of about 0.01 µm/s) that coincided with pulsations of the protrusion forming above the sperm head were observed in the fertilized mouse egg; these movements were explained to be caused by contractions of the actomyosin cytoskeleton triggered by Ca^2+^ oscillations induced by fertilization [66]. All the above cells are polarized, contain intracellular electric fields and charged surfaces; therefore, without questioning the existing explanations of the cytoplasmic circulation in these cells, we hypothesize that electroosmotic flow might play an important role in the observed cytoplasmic streaming and should not be ignored.

Another group of cells, where electroosmotic flow might be important, includes neurons and muscle cells where pulses of electric potential (∼100 mV) travel along the cellular membrane, which might carry surface charge (similar to the above models) and therefore support electroosmotic flow complementing and intertwining with the active transport of biomolecules along these cells. Based on the universal nature of electroosmotic flow (the only requirements are charged surface and electric field parallel to this surface), one can expect the role of electroosmosis in the extracellular phenomena as well, especially in the areas of high electric activity, like brain. One of the possible implications is the potential role of electroosmosis in the perivascular transport of extracellular proteins (including amyloid β) out of the brain, where various mechanisms are debated of the fluid movement in the perivascular space in the direction opposite to the blood flow in the artery lumen [68]–[70].

## Conclusions

In this study, we argued that electroosmosis due to the presence of intracellular electric field and charged surfaces might play an important role in the intracellular transport of the macromolecules. This statement was illustrated by two mathematical models of the polarized cells. The first model represented the transport of negatively charged phosphorylated messenger proteins from the cellular membrane to the negatively charged nucleus and demonstrated that the presence of electroosmotic flow could intensify the transport of the messenger proteins to the nucleus and result in the higher (up to 4-fold relative to pure diffusion) and narrower peaks of the messengers' flux on the nucleus. In our model, electroosmosis enables the transport of the negatively charged messenger proteins against the repulsive electrostatic force of the negatively charged nucleus, while in the absence of electroosmosis only small fraction of the messenger molecules would reach nucleus by diffusion against the electric field. Therefore, electroosmosis is shown to be a possible solution to the seemingly paradoxical transport of the negatively charged messenger proteins to the negatively charged nucleus. The second model simulated the process of fluorescence recovery after photobleaching. It was demonstrated that due to the presence of electroosmosis, the time of 50% recovery of the intensity of fluorescence (which is used for determination of the diffusion coefficients of the biomolecules inside the cells) might be dependent (up to 3-fold) on the position of the bleaching sport relative to the cellular membrane. It was suggested that this dependence might explain large run-to-run variability in apparent diffusion coefficient values measured in FRAP experiments.

The developed models are obviously idealizations and simplifications of the real cell structure which includes cytoskeleton, endoplasmic reticulum, mitochondria, and many other organelles. As measured in [4], strong inhomogeneous cytoplasmic electric field is present across the cytoplasm and the organelles of the cell. The surfaces of the organelles are likely charged and therefore can support electroosmotic flows which might contribute to the fast transport of the biomolecules. The presence of the multiple charged surfaces in the intracellular space of the real cells makes electroosmosis likely even more important than in the idealized situation modeled in the current paper. Experimental mapping of the charged surfaces (electrical double layers) together with the mapping of the intracellular electric fields and computation of the electroosmotic flows is an experimentally and computationally intense task that would require collaborative effort of several groups. One of the goals of this paper was to prove that this effort is worth making.

Electroosmotic flow is quite universal; the necessary and sufficient conditions are the charged interface and electric field parallel to this interface. The magnitude of the effect can be very substantial in the biological systems as illustrated by the above models. Therefore, one might speculate on the possible wider implications of electroosmotic flow in the biological processes, including cytoplasmic streaming in plant and embryonic cells and perivascular transport of proteins in brain. The aim of this paper was to draw the attention of the biologists and biophysicists to this important physical phenomenon well known in separation science and microfluidics, however, largely ignored in biology, the main exception being the plant physiology [29]–[35], where electroosmosis was recognized as a mechanism to pump water upward through the phloem.

## Supporting Information

Methods S1 **Parameters Justification file contains an extensive literature review justifying the parameters and assumptions of the models of the above paper.**
(DOC)
