# Supplementary material for: Cytoplasmic Electric Fields and Electroosmosis: Possible Solution for the Paradoxes of the Intracellular Transport of Biomolecules
Source: PLoS One. 2013 Apr 16;8(4):e61884. doi: 10.1371/journal.pone.0061884 (PMC3627925; doi:10.1371/journal.pone.0061884)
Supplement: Methods S1 — Parameters Justification file contains an extensive literature review justifying the parameters and assumptions of the models of the above paper. (DOC) [file pone.0061884.s001.doc]

# S1 Methods. Parameters Justification

## Justification of Model Assumptions and Parameters’ Values

**Cytoplasmic field strength, inward and outward current density.** The experimental paper [s1] reported of the cytoplasmic electric fields in the range of -5∙10^5^÷-3∙10^6^ V/m along the 4.5 µm line in the cytoplasm of living astrocytes. The measurements of [s1] are based on the observation of the fluorescence of the nanoparticles filled with the voltage sensitive fluorescent dye. The larger scale pictures of the astrocytes [s1] demonstrate that although the domains with similar level of fluorescence (i.e. electric field strength) are distributed all across the cytoplasm, the distribution of electric field is non-uniform and likely reflects the presence of multiple charged surfaces including cytoskeleton, endoplasmic reticulum and other organelles. Mathematical modeling of such environment is obviously a very complicated and computationally intense problem far beyond the scope of this study. Our aim is to determine the lower estimate of the role of electroosmosis in the intracellular transport. Therefore, we need to use some reasonable averaged estimate of the electric field strength in the cytoplasm of the polarized cell.

The range of reported values of cytoplasmic electric fields and currents is quite broad. The author of [s2] illustrated his idea of a cell as a “miniature electrophoretic chamber” by presenting sketches of the cylindrical cells with membrane potentials at the opposite walls varying from -36 mV to -40 mV. The study [s3] of the epithelial cells of the snail *Achatina achatina* demonstrated the transepithelial potential difference in the range of -6÷10 mV. The pioneering paper [s4] demonstrated the self-generated electric current of 64∙10^-11^A per cell through the 70 µm diameter of the developing *Fucus furcatus* egg cell which translates into 0.04A/m^2^ current density. Much higher self-generated current of 5∙10^-6^ A per cell was demonstrated in the oocytes of *Hyalophora cecropia* [s5] (30µm diameter cells), which corresponds to the 1800 A/m^2^ current density. Theoretical study [s6] of the ionic permeability of epithelial tissues operated with the value of 50 mV potential difference across epithelia, based on the experimental studies of mammalian small intestine [s7], rabbit gallbladder [s8], and renal proximal and distal tubule [s9].

Based on the above data, and the notion that the potential difference across the polarized cell is generated by the asymmetrical distribution of ion channel and ion pump activity and following [s2, s5], we formulated the boundary conditions by specifying the inward and outward current density at the upper and lower walls of the cylinder (our model of the polarized cell) as *J_n_=* 1500 A/m^2^(equivalent to 1.17∙10^-6^ A per cell), which resulted in potential difference of 49 mV which was in agreement with the range observed in [s6-s9].

**Zeta-potential of the cellular membrane.** The charge of the outer surface of the cellular membrane and the surface potential of the cell were the topics of extensive theoretical and experimental studies as reviewed in [s10]. The theoretical study [s11] analyzed the dependence of the surface potential on biological surfaces on the pH and ionic strength of the solutions and compared it with the values of zeta-potential of the cells measured from the electrophoretic experiments. The values of zeta-potential for human erythrocytes at pH=7 varied from -10mV to -70 mV [s12] and for sheep leucocytes from -10mV to -40mV [s13]. In the experimental study [s14], zeta potential of 3T12 cell at pH7.3 varied from -65mV to -30mV with ionic strength changing from 0.001M to 0.1M. Paper [s15] presented an overview of membrane electrostatics with the emphasis on the biological membranes and comparison of its properties with the artificial lipid bilayer membranes, arguing that “owing to the membrane asymmetry the electrostatic potential at the inner surface can be appreciably higher. For erythrocytes it is believed [s16] to be about -60 mV”.

The experimental paper [s17] studied the electrophoretic mobility and the zeta-potential (-60÷-35mV) of rat liver mitochondria treated with inhibitors and stimulants of mitochondria bioenergetics and compared it with the measured transmembrane potential of these mitochondria. Based on this and other experiments [s18], the authors of [s19] argued that zeta-potential of the cell was strongly affected by the transmembrane potential and was therefore depended on the cellular metabolism. Based on this reasoning they further developed the “ion pump electroosmotic trap” model explaining the experiments [s20-s22] where nanoparticles were reversibly attracted to the living cells but not to the dead cells. The authors of the model predict that “ion pump not only separates electric charges, it also generates surface current along the cell surface; this surface current would involve liquid in motion, similar to the well-known electroosmotic effect” [s19]. By talking about the electroosmotic flow through the ion pump and along the outer surface of the cellular membrane, the authors of the paper [s19] came very close to the anticipation of the intracellular electroosmotic flow; however, they did not discuss it explicitly.

As shown above, there is not much data to estimate the zeta-potential of the inner surface of the cellular membrane. The experimental data for zeta-potential of the outer surface of the cell was in the range -30÷-10mV at the physiological ionic strength 0.1M. Following the reasoning of [s15] that “the electrostatic potential at the inner surface can be appreciably higher”, we assume ζ=-50 mV, which agrees with value (-60 mV) predicted for the inner surface of erythrocytes from the measurements of [s16].

**Viscosity.** Experimental data on the viscosity of cytoplasm is quite controversial. As reviewed in [s23], the 17 papers published from 1950 till 1986 reported the values of cytoplasm viscosity that varied from 0.002 Pa∙s in Swiss 3T3 cells cytoplasm [s24] to 10^4^ Pa∙s in squid axoplasm [s25] (compare to 0.0007 Pa∙s, viscosity of water at 37 ^0^C). The measured values of the cytoplasm viscosity correlate well with the size of the particle probe used in the measurements, (e.g. in [s24] particle diameter was 6∙10^-4^µm while in [s25] it was 83 µm), which agrees with the notion that cytoplasm is a highly inhomogeneous environment composed of aqueous phase, complicated matrix and crowded with organelles, and biomolecules [s26]. The measurements of [s23-s25] therefore present the apparent viscosity which integrated the variety of particle-cytoplasm interactions at the scale of the probe diameter. The more recent review [s27], reported of the cytoplasmic viscosity of 0.001-0.002 Pa∙s estimated through NMR measurement of rotational mobility of phosphoglycerate kinase [s28], and deoxymyoglobin [s29], as well as much higher value of 0.011 Pa∙s estimated from the behavior of two small fluorescent spin probes [s30], and much lower value, i.e. not significantly different from that of water [s31]. The author of [s27] attributed the above high value of viscosity [s30] to the binding of rotational probes to intracellular components and concluded that “average mobility of water in living cells does not appear to be greatly altered compared with pure water, but significant layers of more or less ordered water cannot be ruled out”. Some recent studies [s32-s33], however, again reported of higher cytoplasm viscosity. The value of 0.0045 Pa∙s was estimated for the viscosity of cytoplasm of live *Amoeba proteus* from the experiments in tracking the endogenous particles (radius ~0.5 µm) which unlike exogenous particles could not damage the structure of the cell. The much higher viscosity of 1.38 Pa∙s was estimated for the cytoplasm of drosophila oocytes by using the single point passive microrheology with beads of two sizes (diameter 0.5 µm and 1 µm) and two different coatings to ensure that “beads did not stick to any proteins” [s33].

The controversial data on the viscosity of cytoplasm and its dependence on the size of the probe particles, i.e. on the scale of spatial averaging, make it difficult to select the value of viscosity for our model of intracellular electroosmotic flow. Given the above information, we selected the intermediate viscosity value of 0.002 Pa∙s determined with ultra small probe particles [s24] and consistent with the results of the recent studies [s28, s32]. The value of viscosity is important for evaluation of the magnitude of the electroosmotic flow velocity; however, as shown in the Discussion section, it cancels out in the comparison of the relative impact of diffusion and electroosmosis in the transport of biomolecules, when the Einstein-Stokes equation (s1) for diffusion coefficient is used.

**Diffusion coefficient of proteins in cytoplasm.** Diffusion coefficient and viscosity of the medium are closely related. Diffusion coefficient in the homogeneous medium is determined by the Einstein-Stokes equation:

$D=\frac{k_{B}T}{6\pi\eta r_{S}}$ (s1),

where *η-* viscosity of the medium and *r_S_* –Stokes (or hydrodynamic) radius of the particle. In the inhomogeneous environment like cytoplasm, the situation is more complicated. As stated in [s34], “objects smaller than about 500 kDa diffuse freely in the cytoplasm, while objects larger than about 20 nm are macroscopically immobile due to the high viscosity of the cytosol and the presence of a dense meshwork of cytoskeletal filaments “.

According to the theory proposed in [s35], diffusion coefficient of a small solute in cytoplasm is reduced relative to its diffusion coefficient in water *D_o_* due to three independent factors:

$D/D_{0}=F_{1}\cdot F_{2}\cdot F_{3}$ (s2),

where factor $F_{1}=\eta_{0}/\eta$is simply the ratio of viscosities of water and cytosol, while *F_2_* and *F_3_* describe the effects of binding and steric hindrance. The model developed in [s26] continued this approach and argued that *F_2_* could be modeled from the known or estimated direct and reverse binding constants, while *F_3_* needed to be determined from the experimental data and depended not only on the size of diffusing molecule, but on the size of the cell and its organelles as well. According to [s26], the value of *F_3_* varied from 0.4 for the molecule with 0.59 nm radius in the cell of 87 µm radius to 0.93 for the molecule with 0.57 nm radius in the xenopus oocyte cell of 1000 µm radius.

According to experimental results reviewed in [s27], the ratio *D/D_o_* for the inert fluorescent tracers declined “in cultured *Xenopus* neurons from 0.39 to 0.05 for particles ranging from 2 to 44.8 nm hydrodynamic radius” [s36] and “in skeletal muscle myotubes, from 0.4 to 0.13 for particles ranging from 2.9 to 12.6 nm” [s37]. These results are consistent with the model of [s38] where diffusion is restricted due to sieving by a network of cytoskeleton filaments. Note that the value of *D/D_o_=*0.3÷0.4 for the small inert tracer particle (likely not influenced by the steric factor) is consistent with the *η/η_0_*=2.86 selected for the ratio of viscosities of cytoplasm and water in our study (*η=*0.002 Pa∙s).

The apparent diffusion coefficients determined by FRAP experiments in intact cells (14 studies of *D/D_o_* for various proteins reviewed in [s27]) are much lower than predicted and measured for the inert particles of similar size which is most likely due to binding interactions with intracellular components (factor *F_2_* ). In general, the value of *D/D_o_* is not correlated with the radius of the protein and differs from 0.007 for insulin (*r_s_*=1.6 nm) [s39] to 0.31 for green fluorescent protein (*r_s_*=2.5 nm) [s40]; the later shown to have very few ionizable or hydrophobic groups on its surface, which explains its apparent lack of binding [s41].

The recent paper [s42] introduced a new method of mesoscopic modeling of protein diffusion in the cell and applied it for analysis of the FRAP data and estimation of diffusion coefficient of enhanced yellow fluorescent protein (EYFP) in two cell lines, fibroblast-like Norden Laboratory Feline Kidney (NLFK) and cervical carcinoma HeLa cells. According to [s42], the diffusion coefficient of EYFP was equal to 15.5±2.7 µm^2^/s in the NLFK and 20.6±5.0 µm^2^/s in the HeLa cells.

In our study, we aim to estimate the possible role of electroosmosis in the transport of messenger proteins from the cellular membrane to the nucleus. Following [s43-s44], we modeled the transport of proteins MEK and ERK. The molecular weight of these proteins is 45 kDa and 44 kDa respectively. The model of [s44] assumed the diffusion coefficient of 50.5 µm^2^/s for these proteins; however this value seems overestimated in the view of the above data. To the best of our knowledge, there are no direct measurements of diffusion coefficients of MEK and ERK in the cytoplasm. Below we present our reasoning for approximate estimation of *D* for these proteins. Following [s45], the stokes radius for the 45 kDa protein is about 3.1 nm, which corresponds to the diffusion coefficient in water *D_0_=*101 µm^2^/s. $F_{1}=\eta_{0}/\eta=0.35$; $F_{2}=1,$ since following [s43-s44] we assumed that messenger proteins do not bind to any cytoplasm objects. We assumed $F_{3}$≤0.4, following the results of [s26] for the molecule with 0.59 nm Stokes radius in the cell of 87 µm radius, based on the reasoning that our molecule is larger (3.1nm) and our cell of interest is much smaller (5 µm radius). Therefore, diffusion coefficient of the messenger proteins in cytoplasm was estimated as $D=D_{0}\cdot F_{1}\cdot F_{2}\cdot F_{3}\leq14.1$µm^2^/s, which is consistent with the results of the FRAP measurements of diffusion coefficients (15.5 µm^2^/s and 20.6 µm^2^/s) of smaller protein EYFP (27kDa) in NLFK cells and HeLa cells (10.5 µm radius [s46]); it is also consistent with the diffusion coefficient of *D=*10 µm^2^/s assumed for signaling proteins in the VCell model of [s47].

**Diffusion coefficient of the proteins in the nucleoplasm.** The exact values of the diffusion coefficients of the proteins inside the nucleoplasm are not too important for our study, since we are interested in the protein transport through cytoplasm and not in its interactions with other proteins (e.g. transcription factors) inside the nucleus. The existing experimental data for the diffusion coefficient of the EYFP protein inside the nucleus (which is supposed not to have binding targets there) of NLFK cells and HeLa cells is much more uncertain than for the cytoplasm, i.e. 18.9±10.8 mm^2^/s (NLFK) and 17.2±13.4 mm^2^/s (HeLa) [s46]. However, the diffusion coefficients of the transcription factor NF-κB in the nucleus and of species bonded to this transcription factor are assumed much lower, i.e.0.0425 µm^2^/s, in the spatio-temporal model of the intracellular signaling pathway [s48]. We use the later value for the apparent diffusion coefficient of our messenger proteins inside the nucleus, since messenger proteins are supposed to interact with the transcription factors in the nucleus and since the detailed investigation of the fate of the protein after it reaches the nucleus is beyond the scope of this paper. As seen in the Results section, our main interest is the total flux of the messenger proteins through the nuclear-cytoplasm interface.

## Assumption of the slip boundary condition

The no-slip boundary condition typically used at the liquid-solid interface has been debated since the 19^th^ century as reviewed in [s49-s50]. The alternative linear slip boundary condition was proposed by Navier [s51] and Maxwell [s52] in the following form:

$\begin{matrix} \mathbf{u}\cdot\mathbf{n}=0 \\ \mathbf{u}_{t}=\lambda\mathbf{n}\cdot(\nabla\mathbf{u}+(\nabla\mathbf{u})^{T})\cdot(1-\mathbf{nn}) \end{matrix}$ (s3),

meaning that the normal component of the velocity is equal to zero, while the tangential component of velocity is proportional to the shear rate at the surface; parameter$\lambda$is called slip length and can be interpreted as the fictitious distance below the surface where the no-slip boundary condition would be satisfied. Since $\lambda$is small compared to channel size in the majority of the problems, it was not important whether to use no-slip or slip boundary condition. However, this is not the case in the narrow channels, e.g. in microfluidics. The more recent studies showed that the no-slip boundary condition is “just one of many allowable flow boundary conditions ranging from pure slip to multi-layer locking. The degree of slip at the boundary depends on a number of interfacial parameters including the strength of the liquid–solid coupling, roughness of the interface, and the commensurability of wall and liquid densities ”[s53- s55]. The situation is even more complicated when the fluid is in contact with the biological surfaces, as reviewed in [s56], where the modifications of the no-slip boundary condition on the macroscopic “riblet” and “hairy” surfaces are discussed. The boundary condition on the cellular and nuclear membranes are even more difficult to define, since depending on the scale it could be viewed both as “hairy” surfaces and as multilayered viscoelastic structures [s57], where deformation and movement of layers is possible. Based on the above experimental and theoretical studies we concluded that the no-slip boundary condition would be too restrictive and would not properly describe the interface between cytoplasm and cellular and nuclear membranes. Instead, we used the slip boundary condition implemented in the COMSOL Multiphysics software and formulated as:

$$\begin{matrix} \mathbf{u}\cdot\mathbf{n}=0, \mathbf{K}-(\mathbf{K}\cdot\mathbf{n})\mathbf{n}=0 \\ \mathbf{K}=(\nabla\mathbf{u}+(\nabla\mathbf{u})^{T})\mathbf{n} \end{matrix}$$

expressing that there is no flow across the boundary and no viscous stress in the tangential direction.

## References for Supporting Information

s1. Tyner KM, Kopelman R, Philbert MA (2007) “Nanosized voltmeter” enables cellular-wide electric mapping. Biophys J 93: 1163-1174.

s2. De Loof A (1985) The cell as a miniature electrophoresis chamber. Comp Biochem Physiol 80A: 453-459.

s3. Tyrakowski T, Holynska I, Lampka M, Kaczorowski P. (2006) The electrical potential difference through the foot epithelium of the snail *Achatina achatina, Lameere* during mechanical and chemical stimulation. Folia Biologica 54: 117-125.

s4. Jaffe LF (1966) Electrical currents through the developing *Fucus* egg. Proc Natl Acad Sci USA 56:1102-1109.

s5. Woodruff RI, Telfer WH (1974) Electrical properties of ovarian cells linked by intracellular bridges. Ann N Y Acad Sci 238: 408-419.

s6. Schultz SG, Frizzell RA (1976) Ionic permeability of epithelial tissues. Biochim Biophys Acta 443: 181-189.

s7. Schultz SG. (1976) Transport across small intestine. In: Tosteson DC, Giebisch G, Ussing HH (editors). Handbook of Biological Membranes. Springer-Verlag, Berlin.

s8. Frizzell RA, Schultz SG (1972) Ionic conductances of extracellular shunt pathway in rabbit ileum: Influence of shunt on transmural Na transport and electrical potential differences. *J Gen Physiol* **59**:318–346.

s9. Windhager EE, Giebisch G. (1965) Electrophysiology of the nephron. Physiol. Rev 45: 214-244.

s10. Andelman D. (1995) Electrostatic properties of membranes: the Poisson-Boltzmann theory. In: Lipowsky R, Sackmann E editors. Handbook of Biological Physics, v1, p 603-641, Elsevier.

s11. Prieve DC, Ruckenstein E (1976) The surface potential of and double-layer interaction force between surfaces characterized by multiple ionizable groups. J Theor Biol 56:205-228.

s12. Heard DH, Seaman GVF (1960) The influence of pH and ionic strength on the electrokinetic stability of the human erythrocyte membrane. J Gen Physiol 43:635-654.

s13. Wilkins DJ, Ottewill RH, Bangham AD (1962) On the flocculation of sheep leucocytes. Electrophoretic studies. J Theor Biol 2:165-167.

s14. Van Wagenen RA, Andrade JD, Hibbs JB (1976) Streaming potential measurements of biosurfaces. J Electrochem Soc 123:1438-1444.

s15. Cevc G (1990) Membrane electrostatics. Biochim Biophys Acta, 1031-3:311-382.

s16. Heinrich R, Gaestel M, Glaser R. (1982) The electric potential profile across the erythrocyte membrane, J Theor Biol 96: 211-231.

s17. Kamo N, Muratsugu M, Kuhinara K, Kobatake Y. (1976) Change in surface charge density

and membrane potential of intact mitochondria during energization. FEBS Lett 72(2): 247–250.

s18. Tsoneva J, Tonov TJ.(1984) Relationship between the power of energization and electrophoretic mobility of rat liver mitochondria. Bioelectrochem Bioenerg 12: 232-258.

s19. Dukhin AS, Ulberg ZR, Karamushka VI, Gruzina TG (2010) Peculiarities of live cells’ interaction with micro- and nanoparticles. Adv Colloid Interface Sci 159: 60-71.

s20. Pohl HA, Braden T, Robinson S, Piclardi J, Pohl DG. (1981) Life cycle alternations of the micro-dielectrophoretic effects in cells. J Biol Phys 9:133–154.

s21. Derjaguin BV, Golovanov MV.(1984) On long-range forces of repulsion betweenbiological cells. Colloids Surf 10:77–84.

s22. Ulberg ZR, Karamushka VI, Vidybida AK, Serikov AA, Dukhin AS, et al. (1992) Interaction of energized bacteria cells with particles of colloidal gold: peculiarities and kinetic model of the process. Biochim Biophys Acta 1134:89–95

s23. Valberg PA, Feldman HA. (1987) Magnetic particle motions within living cells. Measurement of cytoplasmic viscosity and motile activity. Biophys J 52:551-561.

s24. Mastro AM, Babich MA, Taylor WD, Keith AD (1984) Diffusion of a small molecule in the cytoplasm of mammalian cells. Proc. Natl. Acad. Sci. USA. 81: 3414-3418.

s25. Sato M, Wong TZ, Brown DT, Allen RD (1984) Rheological properties of living cytoplasm: a preliminary investigation of squid axoplasm (Loligo pealei). Cell Motil. 4: 7-23.

s26. Chang H-C, Lin Y-C, Kuo C-T (2008) A two-dimensional diffusion model quantifying intracellular transport with independent factors accounting for cytosol viscosity, binding and steric hindrance. Biochemical Engineering Journal 41:217-227.

s27. Luby-Phelps K. (2000) Cytoarchitecture and physical properties of cytoplasm: volume, viscosity, diffusion, intracellular surface area. In: Int Rev Cytol 192: 189-221.

s28. Williams SP, Haggle PM, Brindle KM (1997) F-19 NMR measurements of the rotational mobility of proteins in vivo. Biophys J 72: 490-498.

s29. Livingston DY, LaMar GN, Brown WD (1983) Myoglobin diffusion in bovine muscle. Science 220: 71-73.

s30. Lepock JR, Cheng KH, Campbell SD, Kruuv J (1983) Rotational diffusion of tempone in the cytoplasm of Chinese hamster lung cells. Biopys J 44:405-412.

s31. Fushimi K, Verkman AS (1991) Low viscosity in the aqueous domain of cell cytoplasm measured by picoseconds polarization microfluorimetry. J Cell Biol 112:719-725.

s32. Rogers SS, Waigh TA, Lu JR (2008) Intracellular microrheology of motile amoeba proteus. Biophys J 94:3313-3322.

s33. Ganguly S, Williams LS, Palacios IM, Goldstein RE (2012) Cytoplasmic streaming in drosophila oocytes varies with kinesin activity and correlates with the microtubule cytoskeleton architecture. Proc. Natl. Acad. Sci. USA 109 (38):15109-15114.

s34. Greber UF, Way M (2006) A superhighway to virus infection. Cell 124:741-754.

s35. Kao HP, Abney JR, Verkman AS (1993) Determinants of the translational mobility of a small solute in cell cytoplasm J Cell Biol 120:175-184.

s36. Popov S, Poo MM (1992) Diffusional transport of macromolecules in developing nerve processes. J Neurosci 12: 77-85.

s37. Arrio-Dupont M, Cribier S, Foucault G, Devaux PF, Dalbis A (1996) Diffusion of fluorescently labeled macromolecules in cultured muscle cells. Biophys J 70: 2327-2332.

s38. Hou L, Lanni F, Luby-Phelps K (1990) Tracer diffusion in F-actin and Ficoll mixtures. Toward a model for cytoplasm Biophys J 58:31-43.

s39. Jacobson K, Wojcieszyn J (1984) The translational mobility of substances within the cytoplasmic matrix. Proc. Natl. Acad. Sci. USA 81:6747-6751.

s40. Swaminathan R, Hoang CP, Verkman AS (1997) Photobleaching recovery and anisotropy decay of green fluorescent protein GFP-S65T in solution and cells: cytoplasmic viscosity probed by green fluorescent protein translational and rotational diffusion. Biophys J 72:1900-1907.

s41. Yang F, Moss LG, Phillips GN (1996) The molecular structure of green fluorescent protein. Nat Biotechnol 14:1246-1251.

s42. Kuhn T, Ihalainen TO, Hyvaluoma J, Dross N, Willman SF, et al (2011) Protein diffusion in mammalian cell cytoplasm. PLoS ONE 6(8): e22962.

s43. Gatenby RA, Frieden BR (2010) Coulomb interactions between cytoplasmic electric fields and phosphorylated messenger proteins optimize information flow in cells. PLoS One 5(8): e12084.

s44. Cunningham J, Estrella V, Lloyd M, Gillies R, Frieden BR, et al (2012) Intracellular electric field and pH optimize protein localization and movement. PLoS One 7(5): e36894.

s45. Erickson HP. (2009) Size and shape of protein molecules at the nanometer level determined by sedimentation, gel filtration, and electron microscopy. In: Li S (ed.) Biological Procedures Online, v 11, #1, p 32-51. DOI: 10.1007/s12575-009-9008-x.

s46. Zhao L, Kroenke CD, Song J, Piwnica-Worms D, Ackerman JJ, et al. (2008) Intracellular water-specific MR of microbead-adherent cells: the HeLa cell intracellular water exchange lifetime. NMR Biomed. 21(2):159-64.

s47. Cowan AE, Moraru II, Schaff JC, Slepchenko BM, Loew LM. (2012) Spatial modeling in cell signaling networks. In: Methods in Cell Biology v110, Elsevier Inc, p 195-221.

s48. Terry AJ, Chaplain MAJ. (2011) Spatio-temporal modeling of the NF-κB intracellular signaling pathway: the roles of diffusion, active transport, and cellular geometry. J Theor Biol 290: 7-26.

s49. Lauga E, Brenner MP, Stone HA (2005) Microfluidics: the no-slip boundary condition. In: Foss J, Tropea C, Yarin A, editors. Handbook of Experimental Fluid Dynamics: New-York: Springer.

s50. Goldstein S (1969) Fluid mechanics in first half of this century. Ann Rev Fluid Mech 1:1-28.

s51. Navier CLMH (1823) M´emoire sur les lois du mouvement des fluides. M´emoires de l’Acad´emie Royale des Sciences de l’Institut de France, 6: 389–440.

s52. J. C. Maxwell JC (1879) On stresses in rarefied gases arising from inequalities of temperature. *Phil. Trans. Roy.Soc. Lond.* 170:231–256.

s53. Thompson PA, Troian SM. (1997). A general boundary condition for fluid flow at solid surfaces. Nature 389: 360-362.

s54. Thompson PA, Robins MO (1990) Shear flow near solids: epitaxial order and flow boundary conditions. Phys Rev A 41: 6830-6837.

s55. Neto C, Evans DR, Bonaccurso E, Butt H-J, Craig VSJ (2005) Boundary slip in Newtonian liquids: a review of experimental studies. Rep Prog Phys 68:2859-2897.

s56. Bechert DW, Bruse M, Hage W, Meyer R (2000) Fluid mechanics of biological surfaces and their technological application. Naturwissenschaften 87: 157-171.

s57. Bausch AR, Ziemann F, Boulbitch AA, Jacobson K, Sackmann E (1998). Biophysical J 75(4):2038-2049.
